# Attitudes toward fertility and childbearing scale: an assessment of a new instrument for women who are not yet mothers in Sweden

**DOI:** 10.1186/1471-2393-13-197

**Published:** 2013-10-28

**Authors:** Malin Söderberg, Ingela Lundgren, Kyllike Christensson, Ingegerd Hildingsson

**Affiliations:** 1 Institution of Reproductive Health, Department of Women’s and Children’s Health, Karolinska Institute, Retziusväg 13 A, SE-171 77, Stockholm, Sweden; 2 Institute of Health and Care Sciences, The Sahlgrenska Academy at University of Gothenburg, Box 457, Gothenburg, SE-405 30, Sweden; 3 Department of Women’s and Children’s Health, Obstetrics and Gynecology, Uppsala University, Uppsala, SE-751 85, Sweden; 4 Department of Health Sciences, Mid Sweden University, Sundsvall, SE-851 70, Sweden

**Keywords:** Attitudes, Childbearing, Fertility, Scale development, Exploratory factor analysis, Principal component analysis, Young women

## Abstract

**Background:**

Women in high-resource countries often postpone childbearing. Postponed childbearing may lead to increased health risks for both mother and child and may also result in childlessness. Attitudes among men and women about fertility and childbearing have been studied in different phases of fertile life, but instruments that assess attitudes toward fertility and childbearing among women without children are lacking. The aim of this study is to develop and evaluate a specific instrument, the Attitudes toward Fertility and Childbearing Scale (AFCS), to assess and compare attitudes toward fertility and childbearing using a national sample of Swedish women, who are not yet mothers.

**Methods:**

This study reports on the development of a new instrument and was carried out in three steps: (1) Statements were constructed based on two qualitative studies; (2) Data were collected through web-based questionnaires, and (3) Data were analyzed using statistical tests for construct validity with exploratory factor analysis, internal consistency reliability, and comparative statistics. Student’s t-test and analysis of variance (ANOVA) were performed to analyze differences between the components and background characteristics. One hundred and thirty-eight women participated; they were 20–30 years of age, not mothers, and able to read and speak Swedish.

**Results:**

The instrument showed acceptable sample adequacy, factorability, and reliability using Cronbach’s alpha. Three components were revealed, each one representing a specific underlying dimension of the construct: 1) *importance of fertility for the future* (Cronbach’s α, 0.901); 2) *childbearing as a hindrance at present* (Cronbach’s α, 0.908); and 3) *social identity* (Cronbach’s α, 0.805). Women who were students scored higher in *importance of fertility for the future* than did women who were unemployed. Women living in metropolitan areas and larger cities were more likely to score highly in *childbearing as a hindrance at present* than women living in middle-sized cities or in the countryside. Women in the age group from 25–26 agreed to the largest extent with *childbearing as a hindrance at present*.

**Conclusions:**

The instrument shows acceptable factorability and reliability. Three components were found to be the best solution. Further evaluation is necessary.

## Background

Many women in the age group from 20–30 years who are living in high-resource countries postpone childbearing. Studies from these countries report that women wait to have children because they want to establish independence through education, stay active in the labor market [1,2], reach a certain level of maturity, and have a stable partner [3]. When women were asked about the optimal time for motherhood, they cited factors that influenced their decisions such as lacking the 'right’ partner [4], independence, and the fact that fertility decreases with age [5,6]. When asked about the effects of parenthood on their studies, Swedish postgraduate female students perceived problems balancing work and family life [7]. Other reasons given for postponement were efficient forms of contraception, changes in values, gender equity, partner changes, housing conditions, economic uncertainty, and absence of supportive family policies [8]. Reports from Canada and Germany indicate that maternity benefits have had a limited impact on the timing of childbearing [9,10]. Several studies show that in high-resource societies, delayed childbearing has become the norm; negative connotations are associated with young motherhood [6,11,12]. With regard to a woman’s social situation, the ages between 25–35 years are believed to be the most favorable time to give birth to the first child because most women have completed their education and acquired some skills in the labor market [13]. Postponing childbearing further may lead to increased health risks for both mother and child [14-16] and a gap between desired and achieved fertility can result [1,17]. The woman may not be able to have the children she had wished for and may end up childless [18].

Fertility and childbearing attitudes have previously been described by women in different phases of their fertile life and also by women and men with and without children. These reported views include feelings on whether to have children [19], the reasons for having children [20], attitudes about future motherhood, awareness and understanding of fertility [3,4], attitudes toward parenthood during postgraduate studies [7], ambivalence about parenthood [21], and attitudes toward children and career satisfaction [9]. Furthermore, psychological factors involving motivation for parenthood and the family values associated with postponement have been studied [11], as have whether social age limits exist for childbearing [22].

As no current instrument was found that assesses attitudes toward fertility and childbearing among women not yet mothers, our two qualitative studies [23,24] formed the basis for constructing a new instrument. The qualitative studies were based on interviews with 19 women aged 20–30 years, all of whom lacked the experience of motherhood. The interviews were conducted during 2005 and December 2009–June 2010. The first study focused on experiences of fertility and the second focused on thoughts about childbearing. The results showed that fertility was experienced paradoxically in the following manners: fertility was experienced as a power that had to be suppressed; it was experienced in the present and as a future finite possibility; and one’s own fertile responsibility was governed by society [23]. Furthermore, childbearing was seen as limiting a woman’s present freedom and thought of as a future project, a part of female identity, and a conscious standpoint for which the woman wanted to be prepared by creating the best conditions [24].

The aim of the present study was to develop and evaluate a specific instrument, the Attitudes toward Fertility and Childbearing Scale (AFCS). An additional aim was to assess and compare attitudes toward fertility and childbearing in a national sample of Swedish women who were not yet mothers.

## Methods

### Design

This study reports on the development of an instrument designed to assess attitudes toward fertility and childbearing. Empirical indicators [25] were derived from our two previous qualitative studies based on phenomenology and lifeworld hermeneutics [26], which are useful methods for conceptualizing and operationalizing constructs [27]. The instrument was developed through three steps: (1) the construction of statements from two qualitative studies in which the statements were tested using read-aloud/think-aloud methods; (2) data collection by the consecutive recruitment of women visiting contraceptive clinics; and (3) statistical testing for construct validity with exploratory factor analysis, internal consistency reliability, and comparative statistics. Ethics approval was obtained from the Regional Ethic Committee in Stockholm, Sweden, dnr 2011/953-32.

### Development of the instrument

#### ***Step 1: Construction of statements***

Sixty-eight statements were constructed in Swedish, derived from the main results of the previously mentioned qualitative studies [23,24]. Women were asked to assess the statements on a 5-point Likert scale (1 = totally disagree to 5 = totally agree). For instance, statements such as the following were included: *I look forward to one day becoming a mother*; *Childbearing does not fit into my life right now*. No forward/backward translation has been done at this early stage of instrument development.

Socio-demographic background questions (age, civil status, education, occupation, residence, and use of contraceptives) were also added. The socio-demographic variables were categorized as follows: age— <25 years, 25–26 years, and >26 years; civil status—having a partner (married/cohabiting/in a relationship) versus being single; education—university level versus compulsory school/high school; occupation—studying, working, and unemployed; and residence—metropolitan area/large city versus middle-sized city/countryside.

After constructing the first draft of the instrument, cognitive interviewing [28] with read-aloud/think- aloud methods of the content was carried out with three women who met the inclusion criteria for the study. This approach allowed for understanding the statements from the respondent’s perspective rather than that of the researcher. The inclusion criteria were: being 20–30 years old, not being a mother, and being able to read and understand Swedish. The first author (MS) approached each woman individually and informed her repeatedly during the interview to read the statements aloud and indicate aloud every thought that came into her mind. Two interviews were conducted at the university library and one by telephone. (The reason for interviewing one of the women by telephone was her distance from the interview location). The women’s comments were recorded, transcribed, presented, and discussed in the research group. Twelve statements were removed because they were considered unspecific and irrelevant. Questions that were difficult for the respondents to answer (e.g., negatively worded, ambiguous or unclear statements) resulted in adjustments being made. Fifteen statements were amended including two that were negatively worded and therefore reversed, and one statement that was divided into two.

#### ***Step 2: Data collection***

One hundred and sixty-four antenatal and youth clinics in Sweden were approached and asked to recruit women for the study. Swedish antenatal clinics include contraceptive units for women with and without children. Swedish youth clinics provide young people up to 23 years of age with contraceptive guidance, prescriptions and tests for sexually transmitted infections. Thirty-one midwives from the antenatal and youth clinics agreed to participate in the recruitment. These midwives received written information including a short description of the study, the inclusion criteria for participants, and information to be handed out to the woman regarding the voluntary and anonymous nature of her participation. A poster with information about the study was also attached. Midwives from 12 of the 31 participating clinics recruited a total of 178 women during a four-month period. The remaining 19 midwives did not succeed in recruiting women during that period. The 178 women who agreed to participate after receiving information about the study from the midwife provided their email addresses and were sent a link to an online web-based questionnaire. A completed and returned questionnaire was viewed as informed consent. Four women could not be contacted because of incorrect email addresses; 35 women did not answer the questionnaire although reminders were sent out. One hundred and thirty-nine women returned the questionnaire; one woman was excluded because she had a child. In total 138 cases were assessed as valid, resulting in a response rate of 78%.

#### ***Step 3: Data analysis***

Construct validity: To reduce the number of statements and to emphasize clarity an exploratory factor analysis was carried out using principal component analysis (PCA) with Oblimin rotation [29]. Prior to performing PCA, the suitability of data for factor analysis was assessed through the Kaiser-Meyer-Olkin Measure of Sampling Adequacy (KMO), which is suggested to load above 0.6 [30,31], and Bartlett’s test of sphericity [32] for significance (p ≤ .05). The number of retained components was guided by three decision rules: Kaiser’s criterion (eigenvalues > 1), Catell’s scree test [33] with inspection of the scree-plot, and the use of Horn’s parallel analysis [34]. All components with eigenvalue >1 and statements loading above 0.40 were retained.

Internal consistency reliability: The internal consistency reliability was measured using a Cronbach’s alpha coefficient [35]. With regard to developing a new instrument, the limitation value for alpha was chosen to be more than 0.70 [36]. The effect size was calculated using eta squared to show the relative magnitude of the differences between means [37]. Eta squared ranges from 0 to 1 and represents the proportion of variance in the dependent variable explained by the independent variable [29]. Mean scores and standard deviations were calculated for each of the socio-demographic background variables and each subscale, using ANOVA or Student’s t-test. Higher mean scores indicate higher agreement.

Statistical analysis was conducted using the Statistical Package for Social Sciences, SPSS Version 20 (SPSS, Inc., Chicago, USA). The software developed by Watkins [38] was used for the parallel analysis.

## Results

Fifty-seven statements of the AFCS instrument were subjected to principal component analysis. The Kaiser-Meyer-Olkin value was 0.807 and Bartlett’s test of sphericity reached statistical significance, supporting the sample adequacy and the factorability of the correlation matrix. After examining Catell’s scree test, *three components* were retained, a decision supported by the results of the parallel analysis, which showed three components with eigenvalues exceeding the corresponding criterion values for a randomly generated data matrix of the same size. Solutions with two and four components were subjected to analysis, but the three-component solution with Oblimin rotation proved to be the best and most easily interpreted. To improve and refine the scale, 18 statements with low (< 0.3) communality values were removed. Six statements that were too highly correlated (> 0.8) were dropped. A PCA with 33 statements and a three-component solution showed that individual components explained 28%, 20%, and 5%, or 53% of the total variance. Three statements with double loadings in the pattern matrix were subsequently dropped.

Cronbach’s coefficient alpha was used to assess the internal consistency reliability for each component and ranged from 0.805 to 0.908. To make sure that all of the statements were contributing to high reliability, components that lowered the coefficient alpha value were excluded. Finally, the instrument consisted of 27 statements comprising the three components. The components’ internal consistency reliability was: *Component 1*, nine statements (mean score 35.7, SD 7.8, Cronbach’s α, 0.901); *Component 2*, twelve statements (mean score 42.0, SD 10.9, Cronbach’s α, 0.908); and *Component 3*, six statements (mean score 22.1, SD 4.9, Cronbach’s α, 0.805) (see the pattern and structure matrix presented in Table 1). Following this first analysis of the instrument, the three components were labeled: *importance of fertility for the future* in which children were seen as an essential part of life, a part of personal development, and something to look forward to; *childbearing as a hindrance at present*, where children were considered to limit one’s personal life and imply a responsibility that did not fit the woman’s current life situation; and *social identity*, such as family values and the importance of fertility to a woman’s identity.

**Table 1:** **Pattern and structure matrix of** “**three component**” **PCA solution with Oblimin rotation of attitudinal statements**

**Statements**	**Components**						
	**Importance of fertlity for the future**		**Childbearing as a hindrance at present**		**Social identity**		**Communalities**
	**Pattern coefficients**	**Structure coefficients**	**Pattern coefficients**	**Structure coefficients**	**Pattern coefficients**	**Structure coefficients**	
**I look forward to one day becoming a mother**	**0**.**871**	**0**.**870**	-0.130	-0.312	0.066	-0.286	.779
**Having children is an essential part of life**	**0**.**849**	**0**.**853**	0.051	-0.116	-0.034	-0.395	.732
**Having children will develop me as a person**	**0**.**818**	**0**.**691**	0.170	-0.016	0.221	-0.138	.532
**I find it hard to imagine living a life without children**	**0**.**779**	**0**.**779**	-0.129	-0.291	0.060	-0.253	.629
**I can imagine being pregnant and giving birth**	**0**.**778**	**0**.**846**	-0.079	-0.223	-0.125	-0.443	.731
**Having a child is a way for me to add new elements in life**	**0**.**708**	**0**.**752**	0.110	-0.016	-0.158	-0.456	.603
**I talk to my friends about having children in the future**	**0**.**649**	**0**.**712**	-0.124	-0.245	-0.092	-0.351	.524
**It is important for me to be fertile**	**0**.**632**	**0**.**726**	0.062	-0.039	-0.253	-0.524	.589
**It is important for me to be able to get pregnant anytime**	**0**.**424**	**0**.**579**	-0.189	-0.246	-0.281	-0.439	.416
**Having children would limit my life right now**	0.022	-0.218	**0**.**882**	**0**.**862**	0.150	0.052	.763
**An unplanned pregnancy would hinder me in my current life**	0.019	-0.173	**0**.**801**	**0**.**789**	0.075	-0.014	.628
**Childbearing does not fit into my life right now**	-0.098	-0.271	**0**.**790**	**0**.**806**	0.035	-0.004	.663
**Taking responsibility for a child does not fit into my current life**	-0.130	-0.238	**0**.**782**	**0**.**819**	-0.117	-0.141	.688
**Having children would limit my leisure time activities**	-0.096	-0.254	**0**.**715**	**0**.**731**	0.034	0,002	.548
**I do not want to take responsibility as a mother now**	-0.228	-0.335	**0**.**707**	**0**.**761**	-0.083	-0.059	.620
**Having children would limit my career**	0.092	-0.060	**0**.**687**	**0**.**665**	0.033	-0.075	.449
**Being a mother would take too much of my own time**	-0.319	-0.418	**0**.**626**	**0**.**697**	-0.065	0.006	.569
**Having children would limit my study opportunities**	0.169	0.068	**0**.**623**	**0**.**595**	-0.057	-0.191	.393
**Having children would limit socializing with my friends**	0.043	-0.106	**0**.**600**	**0**.**584**	0.068	-0.010	.345
**It is important for me to choose when to get pregnant**	-0.007	-0.044	**0**.**595**	**0**.**616**	-0.196	-0.253	.416
**It is important for me to have my own stable economy when I have children**	0.003	0.069	**0**.**484**	**0**.**523**	-0.390	-0.440	.425
**My fertility makes me feel communion with other women**	-0.070	0.286	-0.158	-0.066	-**0**.**774**	-**0**.**729**	.555
**Being fertile is important for my identity as a woman**	0.158	0.417	0.099	0.134	-**0**.**667**	-**0**.**743**	.574
**It is important to me that the child is born in a nuclear family ie mother**, **father**, **children**	0.013	0.247	0.139	0.199	-**0**.**626**	-**0**.**646**	.435
**When I have children**, **my life must be prepared for living with children**	0.058	0.218	0.398	0.444	-**0**.**574**	-**0**.**638**	.555
**It is important for me to have a stable relationship when I have children**	0.056	0.276	0.041	0.085	-**0**.**546**	-**0**.**573**	.332
**Becoming a mother is important for my identity as a woman**	0.381	0.583	0.062	0.038	-**0**.**513**	-**0**.**679**	.573

*Note*: Bold values indicate major loadings.

### Socio-demographic background in relation to the subscales

The socio-demographic characteristics of the sample of 138 women showed a population in which 70% had a university education, 32% were studying at university level, and 65% were living in metropolitan areas or large cities. Approximately 75% had a partner and 93% used contraceptives. The mean age was 24.6, with 57% of the women in the age group <25 years. A one-way between-groups ANOVA was conducted to explore the impact of age and occupation on the three components measured by the AFCS (Table 2). In the component *importance of fertility for the future*, there was a statistically significant difference in the scores regarding occupation. The effect size was medium (eta squared = .06). Students were significantly more likely to agree with *importance of fertility for the future* than were the unemployed women. In the component *childbearing as a hindrance at present*, there was a statistically significant difference in scores for the age groups. The effect size was medium (eta squared = 0.09). Women aged 25 or younger were significantly more likely to agree with *childbearing as a hindrance at present* than women in the group >26 years. An independent t-test was conducted to compare component scores with civil status, education, residence, and contraceptive use (Table 2). Women living in metropolitan areas and large cities showed higher levels of agreement with *childbearing as a hindrance at present* than did women living in middle-sized cities or in the countryside. The magnitude of the difference in the means (mean difference = -5.5, 95% *CI*: -9.3 to -1.6) was moderate (eta squared = 0.06). For the component s*ocial identity* no differences were found.

**Table 2:** Components in relation to sociodemographic characteristics and contraceptive use

			**Components**					
			**Importance of fertility for the future**		**Childbearing as a hindrance at present**		**Social identity**	
	**Mean age**	**n = 138 n (%)**	**Mean (SD)**	**p-value**	**Mean (SD)**	**p-value**	**Mean (SD)**	**p-value**
**Age**				.58		.00		.65
<25	22.7	78 (56.5)	35.6 (8.3)		43.0 (11.2)		22.4 (5,1)	
25-26	25.5	26 (18.8)	34.6 (7.4)		45.4 (9.1)		22.2 (4.9)	
>26	28.2	34 (24.6)	36.8 (7.0)		36.3 (9.7)		21.4 (4.5)	
**Civil status**				.08		.20		.38
Having a partner	24.8	104 (75.4)	36.4 (7.3)		41.2 (11.1)		22.4 (4.5)	
Single	24.1	34 (24.6)	33.6 (8.8)		44.0 (10.4)		21.4 (6.0)	
**Education**				.59		.77		.13
College/university	25.1	97 (70.3)	35.4 (8.2)		42.1 (10.2)		21.7 (4.7)	
Cumpulsory school/High school	23.3	41 (29.7)	36.2 (6.9)		41.5 (12.5)		23.1 (5.3)	
**Occupation**				.02		.47		.13
Studying	23.3	45 (32.6)	37.9 (6.6)		43.4 (9.8)		23.2 (4.4)	
Employed	25.1	83 (60.1)	35.0 (7.8)		41.0 (11.7)		21.8 (5.1)	
Unemployed	26	10 (7.2)	31.0 (10.0)		43,1 (9.2)		20.2 (4.8)	
**Residence**				.96		.01		.77
Metropolitan area/large city	25.2	89 (64.5)	35.7 (8.0)		43.9 (10.3)		22.2 (5.1)	
Middle sized city/country side	23.5	49 (35.5)	35.6 (7.5)		38.4 (11.3)		22.0 (4.7)	
**Hormonal contraceptives**				.41		.15		.18
Yes	24.5	121 (87.7)	37.1 (6.7)		42.5 (10.8)		22.4 (4.9)	
No	25.5	17 (12.3)	35.4 (7.9)		38.3 (11.6)		20.7 (4.9)	

Independent-samples T-test och ANOVA.

## Discussion

This study developed a new instrument labeled AFCS to measure women’s attitudes toward fertility and childbearing. The instrument showed acceptable sample adequacy, factorability, and reliability. Three components were revealed, each representing a specific underlying dimension of the construct. The sample showed differences in mean scores in occupations for the first component, *importance of fertility for the future*, and differences in age and residential areas for the second component, *childbearing as a hindrance at present*.

For the development of this instrument the concept to be studied was first defined using existential descriptions [25] from Swedish women, 20–30 years of age who were not yet mothers. Statements were constructed from the results of the first two qualitative studies. The direct link the instrument has to women's existential descriptions can be seen as a strength in the assessment of the instrument's validity [39].

Read-aloud/think-aloud methods of the statements in the instrument were used to contribute to a better understanding of how the statements were perceived and understood [28]. This approach can be seen as a strength in the construction of the instrument although the read-aloud/think-aloud procedures are based on subjective assessments. The results were consistent with those from the previous qualitative studies, which constituted the construct [23,24].

The response rate of 78% was fairly high with respect to completing a web-based questionnaire [40]. Nearly 70% of the women in the sample were of reproductive age, either studying at university, or with a university degree already. These characteristics may have contributed to the rate of response because higher response rates are more common among college students and men and women of reproductive age in high-resource countries [41]. The labeling of the three components in this first stage of instrument development must be interpreted with caution; further studies must be undertaken to determine if all component items have been derived and are correctly interpreted [27]. It is important to remember that this is a new instrument using exploratory factor analysis to explore the underlying components of the construct, namely, attitudes toward fertility and childbearing among women who are not yet mothers. The questionnaire was developed in Swedish and used in a sample of Swedish women. Before international use, a forward/backward translation of the questionnaire should be performed.

Differences were found between women in different occupations where students were more likely to agree with the *importance of fertility for the future* than unemployed women. This result may reflect the different life situations of these groups of women. A wish to become a mother in the future was also found in a previous study consisting of university students [3]. Other studies have found that women who delay childbearing emphasize personal economic independence [42]. Unemployment is linked to poorer health among young women [43], which could be related to pessimism about the future [44]. Further, unemployment has previously been linked to postponement of childbearing [45-47]. However, in this study unemployed women were less likely to agree with the *importance of fertility for the future*, which might reflect their pessimism about the future [44]. Despite the Swedish social support system, which includes well-established childcare and parental leave insurance, there still seem to be barriers regarding the timing of childbearing. We found that women from 25–26 years old tended to agree more with *childbearing as a hindrance at present* than those younger than 25 years and over 26 years. The perception of childbearing as a hindrance could reflect why childbearing is delayed. Reasons why Swedish women may delay childbearing have earlier been described as a wish to maintain the unrestricted freedom of single life, to complete studies, and to procure employment [3,19,21,24]. Our results also show that women living in metropolitan areas and larger cities were more likely to agree with *childbearing as a hindrance at present* than women living in middle-sized cities or in the countryside. One explanation for this could be that more young people live in, and move to metropolitan areas or larger cities to study, find jobs, and so on. In addition, postponement of childbearing is more common among women with higher education [8,48]. Moreover, social structures may affect the timing of childbearing [5,8,49]. Thus 'city-life living’ in the context of metropolitan and larger cities in Sweden may be a social structural factor which influences the perception of childbearing as a hindrance and further impacts the timing of childbearing.

In the future it would be interesting to examine not only the psychometric aspects of the instrument in a larger sample of women, but also to conduct a formal translation of the instrument into English to examine its culture-specific aspects.

## Conclusions

The instrument showed acceptable sample adequacy, factorability, and reliability. Three components were found to be the best solution, although interpretation and the labeling of the components might change in a larger study. Each component reached a high level of internal consistency reliability. To generalize the results and increase the instrument’s validity, a study based on a larger population is advised.

## Competing interests

The authors declare that they have no competing interests.

## Authors’ contributions

MS, IL, IH and KC participated in design of the study, analysis and the draft of the manuscript. MS carried out the data collection. All authors read and approved the final manuscript.

## Pre-publication history

The pre-publication history for this paper can be accessed here:

http://www.biomedcentral.com/1471-2393/13/197/prepub
